# Food insecurity threatens health gains in the Western Pacific

**DOI:** 10.1016/j.lanwpc.2024.101255

**Published:** 2024-11-29

**Authors:** The Lancet Regional Health – Western Pacific

During the 16th and 18th centuries, scurvy, a disease caused by insufficient vitamin C intake, affected millions of sailors due to scarcity of fruits and vegetables during long voyages. Three centuries later, a case of a 50-year-old male in Western Australia diagnosed with scurvy as a result of being unable to afford fresh food or take his prescribed supplements has come to light. As such, the cost-of-living pressure leading to rises in food insecurity has created a “perfect storm” for nutritional diseases, which risk health gains in non-communicable disease (NCD) prevention and control in the Western Pacific region.

Food security means having regular physical and economic access to enough safe and healthy food necessary to live a healthy life. Insufficient food access in Asia and the Western Pacific has been amplified by rising costs of food, fertiliser, and fuel, along with supply chain interruptions, currency devaluation, inadequate resources to meet social and economic demands, as well as extreme weather events and political instability in the post-COVID-19 pandemic era. Food safety, nutrition, and security are inextricably connected. Each year, 125 million people suffer from foodborne illnesses in the Western Pacific region, with children under the age of 5 years making up 30% of cases. An estimated 7000 children die each year from consuming unsafe food. As of 2020, a healthy diet is out of reach for 1.8 billion people in Asia and the Pacific. The countries at greatest risk include Laos and Myanmar, having two of the highest food inflation rates in the world (38.8% in Laos and 17.1% in Myanmar).

Food security issues in the Western Pacific region threaten to undermine progress on reducing the double burden of malnutrition, defined as the co-existence of undernutrition and overweight and obesity across a population. Addressing modifiable risk factors, particularly an unhealthy diet, is well acknowledged as a cost-effective strategy to reduce the burden of NCDs. Between 2000 and 2010, several counties in the Western Pacific were making headway to achieve Sustainable Development Goal target 3.4, which aims to reduce premature mortality from NCDs through prevention and treatment by a third by 2030. However, by 2016, progress slowed and even reversed in some countries, particularly Kiribati and Papua New Guinea. Variability in risk factor reduction across the region has slowed progress, especially for obesity and increased prevalence of elevated fasting blood sugar. At present, only New Zealand, South Korea, and Singapore are anticipated to meet the 2030 target. Foods high in salt and sugar are already major dietary sources in some countries in the region, such as Fiji, making behavioural and environmental strategies that aim to promote healthier food choices imperative. Findings from the China Multi-Ethnic Cohort (CMEC) study showed that minority ethnic groups in less developed plateau areas in southwest China had suboptimal diets, linked to increased diet-related cardiometabolic risk. Unfortunately, the current food security situation is not conducive towards helping people make healthier food choices. Even in high-income countries, such as Australia, high food prices, especially for fruits and vegetables, and limited food availability contribute to changes in dietary behaviours. For example, meal skipping among people at increased risk of NCDs such as low-income earners and First Nations Australians.

Four years since the start of the COVID-19 pandemic, signs of health system recovery can be seen worldwide, with a reduction in essential health service disruptions from 56% in 2020 to 23% in 2022. However, compared to pre-pandemic levels, 11% and 7% of countries reported increases in service volumes related to nutrition and NCDs. Approximately half of countries still reported increased backlogs compared to 2021, particularly in screening, diagnosis, and treatment of cancers. In this light, health system and economic recovery could be further optimised by greater focus on policies and interventions that target the food environment and a wider range of lifestyle factors. For example, a non-inferiority, randomised controlled trial of an online lifestyle therapy targeting nutrition and physical activity was found to be as effective as psychotherapy in reducing symptoms of depression and running cost. The development of the National Strategy for Food Security in Remote First Nations Communities is underway to facilitate a coordinated response to improve price, availability, and quality of food and essential groceries for First Nations Australians living in remote areas. Initiatives to enable more sustainable food systems are fundamental, especially given major risk factors for food insecurity, include climate-related shock and reliance on agriculture, pertinent to many countries across the region. Further research that directly examines the relationship between food insecurity and NCDs, which is currently lacking, is vital for effective regional NCD policies and guidelines.

Food security is central to facilitating the adoption of healthy eating habits. A healthier diet has become increasingly out of reach for many people across the Western Pacific region, and further perpetuates social and health inequities. Food insecurity must be prioritised, especially for vulnerable populations, to accelerate healthy lifestyle initiatives and progress in the region.

